# Improvement of D-lactic acid production from methanol by metabolically engineered *Komagataella phaffii* via ultra-violet mutagenesis

**DOI:** 10.1016/j.mec.2025.e00262

**Published:** 2025-05-17

**Authors:** Yoshifumi Inoue, Kaito Nakamura, Ryosuke Yamada, Takuya Matsumoto, Hiroyasu Ogino

**Affiliations:** Osaka Metropolitan University, Department of Chemical Engineering, 1-1 Gakuen-cho, Naka-ku, Sakai, Osaka, 599-8531, Japan

**Keywords:** D-lactic acid, *Komagataella phaffii*, Methanol, Ultra-violet mutagenesis, Transcriptome analysis, Yeast

## Abstract

Methanol has attracted attention as an alternative carbon source to petroleum. *Komagataella phaffii*, a methanol-assimilating yeast, is a useful host for the chemical production from methanol. A previous study successfully constructed a metabolically engineered *K. phaffii* GS115/S8/Z3 strain capable of producing D-lactic acid from methanol. In this study, we aimed to develop a strain with improved D-lactic acid production by applying ultra-violet mutagenesis to the D-lactic acid-producing strain, GS115/S8/Z3. The resulting mutant strain DLac_Mut2_221 produced 5.38 g/L of D-lactic acid from methanol, a 1.52-fold increase compared to the parent strain GS115/S8/Z3. The transcriptome analysis of the mutant DLac_Mut2_221 identified 158 differentially expressed genes, providing insights into key mechanisms contributing to enhanced D-lactic acid production. Metabolic engineering strategies for *K. phaffii* based on the knowledge gained from this study will contribute to improving the productivity of various useful chemicals from methanol.

## Highlights

• UV mutagenesis improved D-lactic acid production in engineered *K. phaffii*.
• The mutant strain produced 1.52 times more D-lactic acid than the parent strain.
• Transcriptome analysis revealed 167 differentially expressed genes in the mutant.
• Three key mechanisms for efficient D-lactic acid synthesis have been revealed.
• The findings of this study are useful for producing compounds from methanol.

## 1 Introduction

Methanol, a C1 compound, continues to undergo advances in manufacturing technology (Deka et al., 2022). Additionally, the synthesis of methanol from CO_2_ and waste biomass is actively progressing, and the methanol market is expected to continue to grow (Schrader et al., 2009; Kelso et al., 2022). These characteristics position methanol as a promising alternative carbon resource to petroleum. Currently, the production of useful compounds using methanol relies mainly on chemical synthesis (Tabibian and Sharifzadeh, 2023). These chemical processes require high-temperature and high-pressure conditions and have low reaction specificity, requiring huge energy and resource costs to produce the products (Burg et al., 2016).

In contrast to chemical processes, bioprocesses have several advantages: they can operate under mild conditions, exhibit high reaction specificity, allow the synthesis of structurally complex compounds and polymers, and allow selective production of chiral compounds (Süntar et al., 2021). Owing to these benefits, research on the production of valuable compounds from methanol using methylotrophic microorganisms has increased (Zhang et al., 2018; Zeng, 2019; Chen and Lan, 2020). Lactic acid is particularly advantageous for bioprocess production. As chiral compounds, both L- and D-forms are utilized in various fields, including the food, cosmetics, pharmaceutical, and chemical industries (Santoro et al., 2016; Jia et al., 2018; Esteban and Ladero, 2018; Beitel et al., 2020; Alexandri et al., 2022). Notably, polylactic acid as a bioplastic has garnered significant attention, with studies reporting improved polymer thermal resistance when equal amounts of poly L-lactic acid and poly D-lactic acid are combined to form a stereocomplex structure (Tsuji, 2005). However, only a limited number of lactic acid bacteria can selectively produce D-lactic acid over L-lactic acid, and there is a need to establish a highly efficient method for producing D-lactic acid (Klotz et al., 2016).

*Komagataella phaffii* (formerly *Pichia pastoris*) is a safe microorganism that has been reported to enable the production of various fine biomolecules through genetic engineering (Çaloglu and Binay, 2023; Ergün et al., 2021; Ergün et al., 2022). *K. phaffii* is capable of metabolizing methanol as its sole carbon source and has been utilized as a host for the production of valuable proteins and compounds through metabolic engineering (Ahmad et al., 2014; Wu et al., 2023; Zhang et al., 2023). Cai et al. constructed a *K*. *phaffii* strain capable of high-level production of free fatty acids (23.4 g/L) and fatty alcohols (2.0 g/L) from methanol by enhancing the acetyl-CoA generation pathway and NADPH supply pathway using CRISPR/Cas9 technology (Cai et al., 2020). Lactic acid can be produced from methanol using methylotrophic yeasts (Yamada et al., 2019; Wefelmeier et al., 2023; Wu et al., 2025). In our previous study, we constructed *K. phaffii* GS115/S8/Z3, in which four copies of the *D-LDH* (D-lactate dehydrogenase) gene derived from *Leuconostoc mesenteroides* were introduced into the rDNA locus under the control of the *AOX1* promoter and *AOX1* terminator, and the strain produced 3.48 g/L D-lactic acid from methanol (Yamada et al., 2019). However, these levels are significantly lower than those produced from glucose (Mitsui et al., 2020; Pangestu et al., 2022). Methanol taken up by the cell is first converted to formaldehyde by alcohol oxidase (AOX), which is then converted to pyruvate via the xylulose-5-phosphate pathway during assimilation metabolism (Jordà et al., 2012; Unrean, 2013). Lactic acid is produced by the conversion of pyruvate by lactate dehydrogenase (LDH). During the dissimilation of methanol in methylotrophic yeasts, formaldehyde is converted to formic acid, which is excreted as CO_2_. To promote the production of methanol-based compounds, suppressing this dissimilation pathway (Guo et al., 2021), avoiding the accumulation of formaldehyde (Zhang et al., 2003; Pedro et al., 2015), and enhancing the metabolic pathways of target compounds have been suggested (Wu et al., 2023). However, methanol metabolism in *K. phaffii* is complex and tightly regulated, and metabolic engineering techniques to efficiently convert methanol into useful compounds are still under development.

Both rational metabolic engineering strategies and random mutagenesis are commonly employed to enhance the productivity of target compounds in microbial metabolism. We have previously optimized *D-LDH* expression to enhance D-lactic acid production in *K. phaffii*, constructing a strain capable of producing 5.18 g/L of D-lactic acid (Inoue et al., 2024). In contrast to metabolic engineering approaches, random mutagenesis introduces random mutations into an organism's genome, facilitating the generation of diverse mutants. Due to the random nature of mutations, previously unreported genes and metabolic pathways may be modified. Random mutagenesis methods include those utilizing the CRISPR-Cas system (Mitsui et al., 2020), ultraviolet (UV) irradiation (Wu et al., 2021), plasma treatment (Kabarkouhi et al., 2024), and chemical mutagens such as ethyl methanesulfonate (EMS) (Prabhu et al., 2020).

UV mutagenesis randomly alters the DNA base pairs in the genome of an organism. UV mutagenesis is a simple method of irradiating organisms with UV radiation, which can damage genomic DNA and introduce mutations during repair. Owing to its simplicity and usefulness, numerous studies on various organisms have used UV irradiation to generate strains with desirable phenotypes (Moshinsky and Wogan, 1997; Guo et al., 2019; Karitani et al., 2024). In *K. phaffii*, UV irradiation improves the production of rFIP-glu protein (Wu et al., 2021). These findings suggest that by introducing UV mutations into *K. phaffii*, it may be possible to obtain mutant strains with enhanced methanol utilization pathways that are intricately regulated.

In this study, we aimed to develop a *K. phaffii* strain capable of producing D-lactic acid from methanol with high efficiency. UV irradiation was performed on the metabolically engineered D-lactic acid-producing strain *K. phaffii* GS115/S8/Z3 (Yamada et al., 2019) to obtain mutant strains with improved D-lactic acid production. Additionally, transcriptome analysis was conducted on the mutant strain to investigate genes related to D-lactic acid production from methanol in *K. phaffii*.

## 2 Materials and methods

### 2.1 Strains and media

The metabolically engineered D-lactic acid-producing strain, *K. phaffii* GS115/S8/Z3 (Yamada et al., 2019) was used in this study. Yeast/peptone/dextrose (YPD) medium containing 10 g/L yeast extract (Formedium, Norfolk, UK), 20 g/L peptone (Formedium), and 20 g/L glucose (Nacalai Tesque, Kyoto, Japan) and yeast/peptone/methanol (YPM) medium containing 10 g/L yeast extract, 20 g/L peptone, and 30 g/L methanol (Nacalai Tesque) were used for cultivation. If necessary, 20 g/L agar (Nacalai Tesque) and 0.1 g/L zeocin antibiotic (InvivoGen, California, USA) were added.

### 2.2 Yeast cultivation

Microplate culture was performed in 1.0 mL of YPM medium using a 2-mL 96-well deep-well plate equipped with a gas-permeable seal (EXCEL Scientific, California, USA) and a rotary plate shaker (Taitec, Nagoya, Japan) set at 30 °C and 1,200 rpm. Cultivation was initiated by culturing the cells in a well containing 1.0 mL of YPD medium at 30 °C and 1,200 rpm for 24 h, harvesting and washing the cells, and suspending them in 1.0 mL of fresh YPM medium.

Flask cultures were performed using a rotary shaker (Taitec) operated at 30 °C, 200 rpm, with 250 mL flasks containing 50 mL of YPM medium equipped with a gas permeable seal (EXCEL scientific). Cultures were started by inoculation (initial OD_600_ of 10.0) of pre-cultures grown in 250 mL flasks containing YPD medium for 24 h at 200 rpm and 30 °C.

### 2.3 Mutagenesis into *K. phaffii*

Yeast cells were cultured in the YPD medium for 24 h. After centrifugation, the cell pellet was resuspended in sterile water to an OD_600_ of 0.5 in a total volume of 10 mL. The suspension was transferred to a petri dish and irradiated with UV light at 18 J/m^2^ for 77 min while stirring. This duration corresponds to the time required to reduce the cell viability to ∼5 % (data not shown). After UV exposure, the suspension was collected and plated onto YPD plates containing 0.1 g/L zeocin. Colonies formed after 72 h of incubation at 30 °C were isolated and maintained on fresh YPD agar plates containing 0.1 g/L zeocin.

### 2.4 Analysis of growth and metabolites

The OD _600_ of each culture was determined using a spectrophotometer (Shimadzu, Kyoto, Japan). D-Lactic acid concentration was calculated using enzymatic reaction method with *D-LDH* (Inoue et al., 2024). Methanol concentration was measured using the previously reported measurement method using 4-amino-3-hydrazino-5-mercapto-1,2,4-triazole (AHMT) (Nacalai Tesque) (Inoue et al., 2024).

### 2.5 Transcriptome analysis

Total RNA was extracted from the cells after 72 h of culture and transcriptome analysis was performed. The cells were collected from the culture broth by centrifugation and disrupted using zirconia beads. Total RNA was isolated from the homogenate using a spin column-type total RNA purification kit (NucleoSpin RNA; Takara Bio, Otsu, Japan) according to the manufacturer's instructions. The MGIEasy RNA Directional Library Prep Set (MGI Tech, Shenzhen, China) was used to prepare a complementary DNA library for next-generation sequencing of the extracted RNA. RNA sequencing was performed using a DNBSEQ-G400 (MGI Tech).

The genome sequences of *K. phaffii* GS115 were used as reference sequences for read mapping using Geneious prime version 2020.0.3 (Tomy Digital Biology, Tokyo, Japan). The differentially expressed genes (DEGs) in the mutant strain were screened using Geneious Prime based on a combined criterion of p-value <0.01 and at least a 1.5-fold change in expression (log2 [fold change] ≥ 0.59 or ≤ −0.59). Gene Ontology (GO) and KEGG pathway enrichment analyses of DEGs were performed using g: Profiler (https://biit.cs.ut.ee/gprofiler/gost). For GO or KEGG pathway enrichment analysis, molecular function, biological processes, and cellular component GO terms or pathway were selected with a p-value threshold of <0.1. RNA sequencing data were deposited in the DDBJ nucleotide sequence database under the accession number PRJDB19698.

## 3 Results

### 3.1 Selection and cultivation of mutant strains generated by UV mutagenesis of GS115/S8/Z3

UV mutagenesis has been used to enhance D-lactic acid production from methanol in yeast. Specifically, UV irradiation was applied to the D-lactic acid-producing yeast strain GS115/S8/Z3, resulting in the isolation of 1,080 colonies, which were designated as DLac_Mut1_1 to DLac_Mut1_1080. These mutant strains were cultured in 1.0 mL of YPM medium using a microplate. Culture supernatants were collected after 48 h of cultivation and D-lactic acid concentrations were measured.

**Fig. 1:**
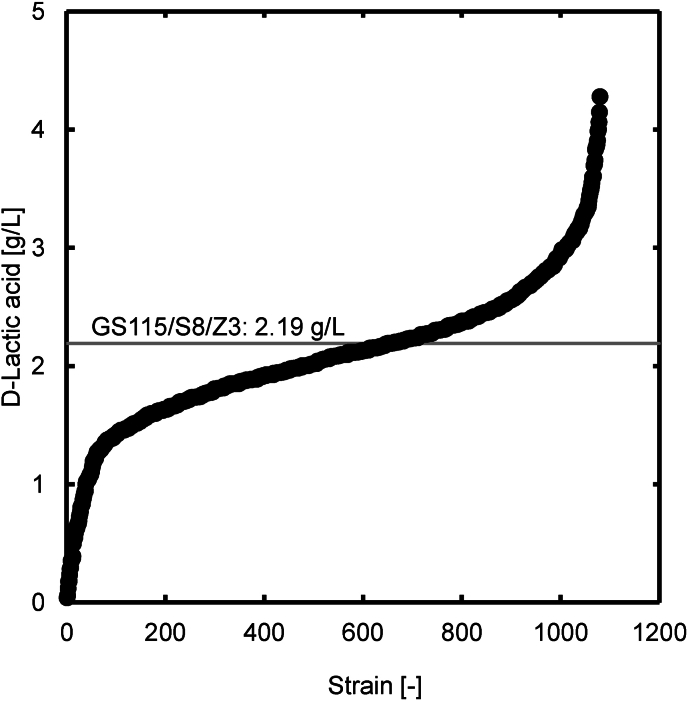
D-Lactic acid concentration after 48-h microplate cultivation of mutants obtained by UV mutagenesis of GS115/S8/Z3. Values are displayed in ascending order from left to right.

The D-lactic acid concentrations of the parent strain GS115/S8/Z3 and the 1,080 mutant strains after 48 h of cultivation are presented in Fig. 1. The D-lactic acid concentration in the parental strain GS115/S8/Z3 was 2.19 g/L. Among the mutant strains, 431 strains showed improved D-lactic acid production. From these 431 strains, the top seven strains with the highest D-lactic acid concentrations were selected: DLac_Mut1_89 (4.28 g/L), DLac_Mut1_372 (4.15 g/L), DLac_Mut1_129 (4.06 g/L), DLac_Mut1_25 (4.00 g/L), DLac_Mut1_260 (3.99 g/L), DLac_Mut1_322 (3.91 g/L), and DLac_Mut1_1077 (3.87 g/L).

**Fig. 2:**
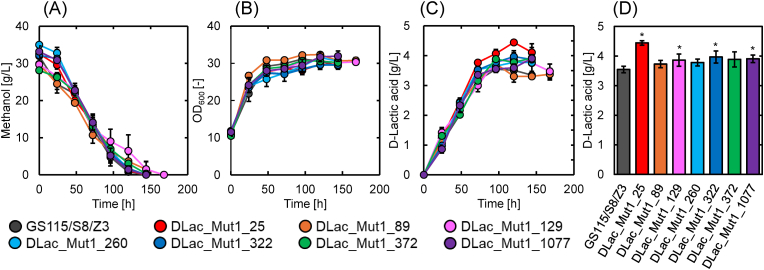
Time-course changes in (A) methanol concentration, (B) OD_600_, (C) D-lactic acid concentration, and (D) maximum D-lactic acid production in GS115/S8/Z3 and seven mutants. Data are presented as the average of three independent experiments. Error bars represent mean ± standard deviation. The statistical significance of the maximum D-lactic acid production compared to the parent strain GS115/S8/Z3 was evaluated using a two-tailed, unpaired, homoscedastic Student's t-test (∗, *p* < 0.05).

Fig. 2 shows the time-course changes in methanol concentration (Fig. 2(A)), optical density (OD_600_) (Fig. 2(B)), and D-lactic acid concentration (Fig. 2(C)) during the flask cultivation of the seven selected mutant strains and the parent strain GS115/S8/Z3. The maximum D-lactic acid production by the seven selected mutant strains and the parent strain is shown in Fig. 2(D). As shown in Fig. 2(A), DLac-Mut1-89 and DLac_Mut1_129 exhausted methanol at 168 h, whereas the other strains depleted it by 144 h. Fig. 2(B) shows that the OD_600_ values for all strains increased significantly up to 48 h of cultivation, reaching ∼30, after which the change in cell density became minimal. There were no substantial differences in cell growth between the parent strain and the seven mutant strains. As shown in Fig. 2(C) and (D), compared with the parental strain, the four mutant strains (DLac_Mut1_25, DLac_Mut1_129, DLac_Mut1_322, and DLac_Mut1_1077) showed statistically significant improvements in D-lactic acid production. Notably, DLac_Mut1_25 achieved the highest D-lactic acid concentration of 4.45 g/L after 120 h of cultivation, representing a 1.25-fold increase compared to the parent strain GS115/S8/Z3.

### 3.2 Selection and cultivation of mutant strains generated by UV mutagenesis of DLac-Mut1-25

To further improve D-lactic acid production, we applied the same mutagenesis procedure to the mutant strain DLac_Mut1_25, which demonstrated enhanced D-lactic acid production after a single mutagenesis (Fig. 2). Overall, 1,080 mutant strains were isolated and designated DLac-Mut2-1 to DLac-Mut2-1080. These mutant strains were cultured in YPM medium (1.0 mL of YPM medium for 48 h using a microplate), and D-lactic acid concentrations were measured.

**Fig. 3:**
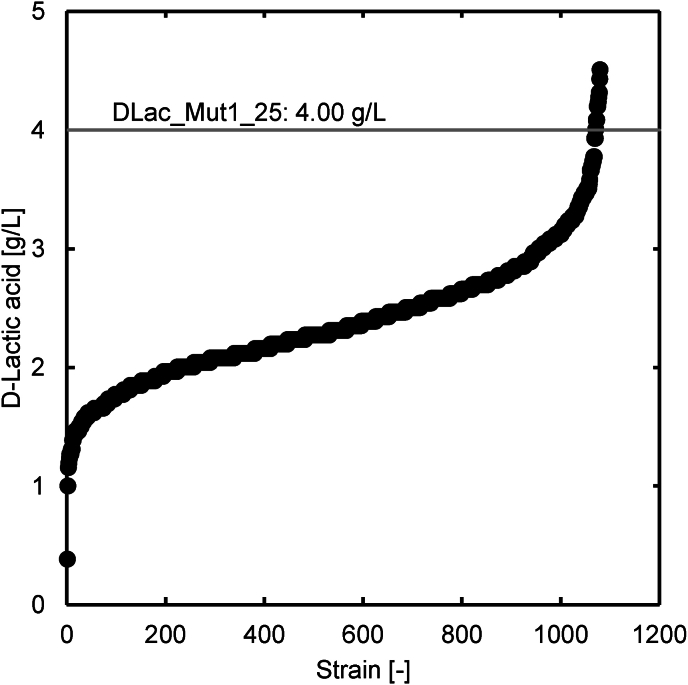
D-Lactic acid concentration after 48-h microplate cultivation of mutants obtained by UV mutagenesis of DLac_Mut1_25. Values are displayed in ascending order from left to right.

D-lactic acid concentrations in the 1,080 mutant strains after 48 h of cultivation are shown in Fig. 3. The D-lactic acid concentration in DLac_Mut1_25 during microplate cultivation was 4.00 g/L. Among the mutant strains, 10 strains exhibited higher D-lactic acid concentrations. The top eight strains were selected: DLac_Mut2_602 (4.50 g/L), DLac_Mut2_741 (4.43 g/L), DLac_Mut2_566 (4.32 g/L), DLac_Mut2_697 (4.28 g/L), DLac_Mut2_946 (4.24 g/L), DLac_Mut2_857 (4.20 g/L), DLac_Mut2_819 (4.20 g/L), and DLac_Mut2_221 (4.09 g/L).

**Fig. 4:**
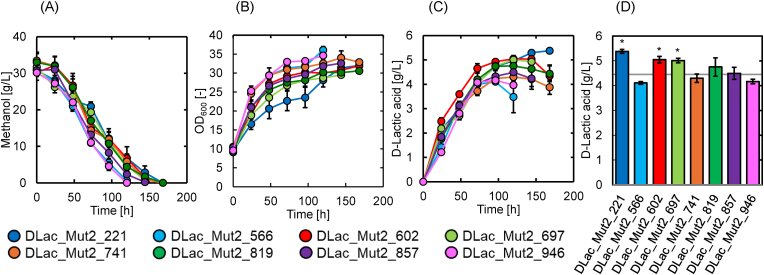
Time-course changes in (A) methanol concentration, (B) OD_600_, (C) D-lactic acid concentration, and (D) maximum D-lactic acid production of eight mutants. Gray line in (D) indicates D-lactic acid production in DLac_Mut1_25 (4.45 g/L). Data are presented as the average of three independent experiments. Error bars represent mean ± standard deviation. The statistical significance of the maximum D-lactic acid production compared to the parent strain DLac_Mut1_25 was evaluated using a two-tailed, unpaired, homoscedastic Student's t-test (∗, *p* < 0.05).

Fig. 4 shows the time-course changes in methanol concentration (Fig. 4(A)), optical density (OD_600_) (Fig. 4(B)), and D-lactic acid concentration (Fig. 4(C)) during flask cultivation of these eight mutant strains. Maximum D-lactic acid production by the eight mutant strains is shown in Fig. 4(D). As shown in Fig. 4(A), methanol depletion occurred at different time points: DLac_Mut2_566 and DLac_Mut2_946 at 120 h; DLac_Mut2_857 and DLac_Mut2_697 at 144 h; and DLac_Mut2_602, DLac_Mut2_741, DLac_Mut2_221, and DLac_Mut2_819 at 168 h. As shown in Fig. 4(B), DLac_Mut2_566 and DLac_Mut2_946 exhibited higher OD_600_ values than the other strains with significantly elevated cell densities after 120 h of cultivation. DLac_Mut2_221 showed lower OD_600_ values until 96 h, but subsequently reached levels comparable to those of the other strains. As shown in Fig. 4(C) and (D), compared to DLac_Mut1_25, three strains (DLac_Mut2_221, DLac_Mut2_602, and DLac_Mut2_697) showed statistically significant improvements in D-lactic acid production. DLac_Mut2_221 produced the highest D-lactic acid concentration of 5.38 g/L after 168 h of cultivation. This represents a 1.21-fold increase compared to DLac_Mut1_25 (4.45 g/L) and a 1.52-fold increase compared to the parent strain GS115_S8/Z3 (3.55 g/L) (Table 1).

**Table 1:** Comparison of D-lactic acid productivity between the parent and mutant strains.

Strain	Relevant features	Maximum D-lactic acid production [g/L]	Fold increase in production^a^^)^ [-]	Culture condition	Reference
Parent strain					
GS115/S8/Z3	Possessing four copies of the pAOX1-*D-LDH*-tAOX1 cassette integrated into the genome	3.48 ± 0.19	0.97	Test tube	Yamada et al. (2019)
		3.55 ± 0.11	1.00	Flask	This study
Mutant strain (single-round mutagenesis)					
DLac_Mut1_25	Obtained by UV mutagenesis of GS115/S8/Z3	4.45 ± 0.08	1.25	Flask	This study
DLac_Mut1_89		3.72 ± 0.10	1.05	Flask	This study
DLac_Mut1_129		3.86 ± 0.21	1.09	Flask	This study
DLac_Mut1_260		3.78 ± 0.11	1.06	Flask	This study
DLac_Mut1_322		3.97 ± 0.20	1.12	Flask	This study
DLac_Mut1_372		3.92 ± 0.48	1.10	Flask	This study
DLac_Mut1_1077		3.91 ± 0.13	1.10	Flask	This study
Mutant strain (two-round mutagenesis)					
DLac_Mut2_221	Obtained by UV mutagenesis of DLac_Mut1_25	5.38 ± 0.08	1.52	Flask	This study
DLac_Mut2_566		4.16 ± 0.06	1.17	Flask	This study
DLac_Mut2_602		5.23 ± 0.13	1.47	Flask	This study
DLac_Mut2_697		4.90 ± 0.09	1.38	Flask	This study
DLac_Mut2_741		4.53 ± 0.17	1.28	Flask	This study
DLac_Mut2_819		4.94 ± 0.37	1.39	Flask	This study
DLac_Mut2_857		4.16 ± 0.25	1.17	Flask	This study
DLac_Mut2_946		4.05 ± 0.09	1.14	Flask	This study

a Relative to production in flask culture of GS115/S8/Z3 strain.

### 3.3 Transcriptome analysis of the D-lactic acid production-improved mutant strain DLac_Mut2_221

**Fig. 5:**
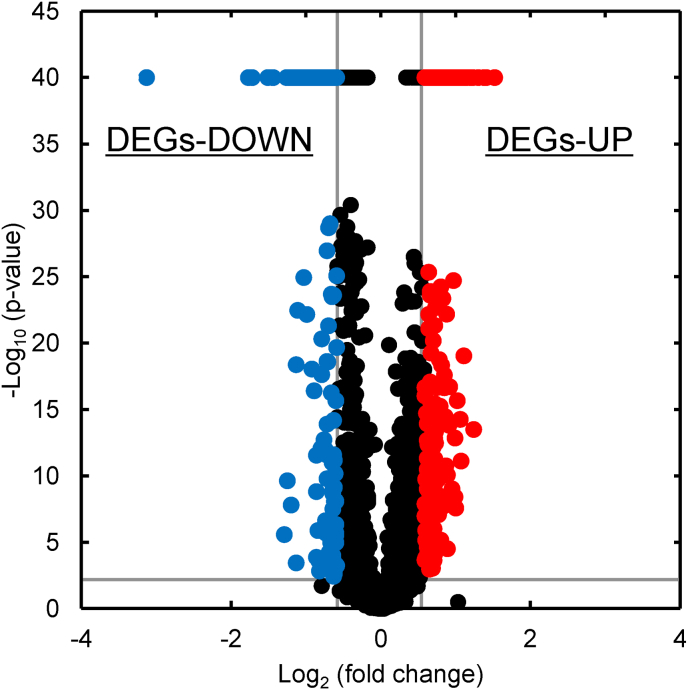
Volcano plot of genes for mutant Dlac_Mut2_221. Vertical lines represent ±1.5-fold change (log_2_ fold change = ± 0.59). Horizontal line indicates a p-value of 0.01 (-log_10_ p-value = 2.0). Red and blue plots represent DEG-UP and DEG-DOWN, respectively, in yeast. Black plots represent non-DEGs (i.e., genes with no change in transcription level).

Transcriptome analysis was performed to elucidate the cause of increased D-lactic acid production in the mutant strain DLac_Mut2_221. The -log_10_ (p-value), which indicates statistical significance, and log_2_ (fold change), which indicates changes in gene expression levels, were calculated for each gene and are shown as volcano plots (Fig. 5). Genes with a high -log10 (p-value) were considered reliable, and genes with a high log_2_ (fold change) showed higher transcription levels in the mutant strain DLac_Mut2_221 than in the parent strain GS115/S8/Z3.

**Fig. 6:**
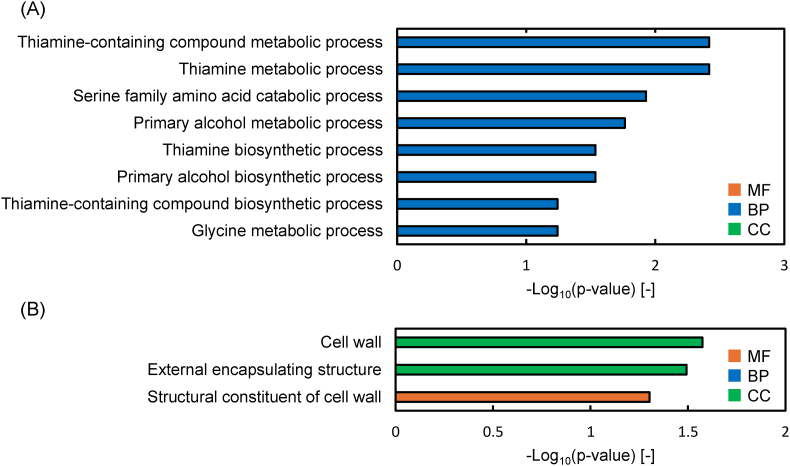
Gene Ontology enrichment analysis. (A) Up-regulated and (B) down-regulated differentially expressed genes. BP, biological process; MF, molecular function; CC, cellular component.

**Fig. 7:**
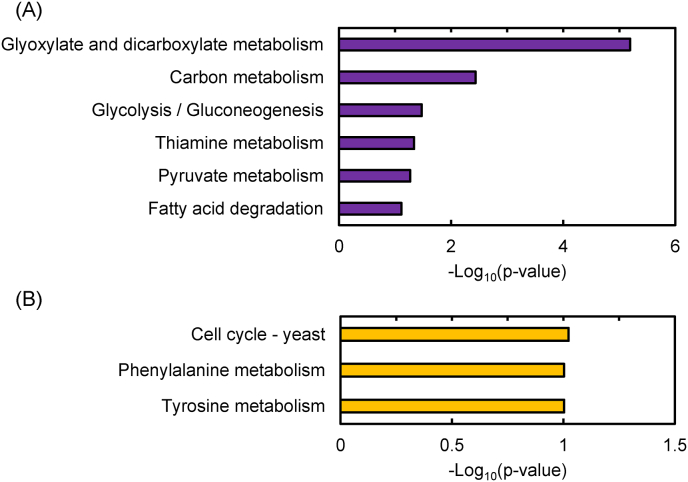
KEGG pathway enrichment analysis. (A) Up-regulated and (B) down-regulated differentially expressed genes.

The transcriptome analysis revealed 158 DEGs in DLac_Mut2_221, comprising 99 upregulated and 59 downregulated genes (Supplementary Table S1and S2). GO and KEGG pathway enrichment analyses of DEGs revealed GO terms that met the threshold p-value of <0.1 (Fig. 6, Fig. 7). Some genes with increased expression (DEGs-UP) were involved in thiamine synthesis and glyoxylate and dicarboxylate metabolism, whereas others with decreased expression (DEGs-DOWN) were involved in cell wall and cell cycle.

## 4 Discussion

Through two-step UV mutagenesis, we obtained the mutant strain DLac_Mut2_221, which produced 5.38 g/L of D-lactic acid (1.52-fold higher than that of the parent strain) after 168 h of culture. (Fig. 4(D)). In our previous study, we successfully produced 5.18 g/L of D-lactic acid from methanol in *K. phaffii* by selecting the *D-LDH* gene, optimizing the expression promoter, and integrating multiple copies into the rDNA locus (Inoue et al., 2024). Sae-Tang et al. also reported that expression of the *D-LDH* gene from *Leuconostoc pseudomesenteroides* and the flocculation gene from *Saccharomyces cerevisiae* in *K. phaffii* improved D-lactic acid production from glucose (Sae-Tang et al., 2023). Wu et al. demonstrated the production of 4.2 g/L L-lactic acid by introducing mutations in the *L-LDH* gene from *Lactobacillus plantarum*, inhibiting lactic acid consumption pathways, and localizing the enzyme to the mitochondria (Wu et al., 2025). Unlike these rational metabolic engineering strategies, the DLac_Mut2_221 mutant strain was generated by simple UV random mutagenesis. To the best of our knowledge, DLac_Mut2_221 achieved the highest concentration of lactic acid, including both D- and L-lactic acid, when utilizing methanol as the sole carbon source. This represents an advancement in D-lactic acid production from methanol using *K. phaffii* and demonstrates the effectiveness of UV mutagenesis as a strategy for strain improvement in *K. phaffii*. The D-lactic acid production with our obtained strain shows a yield of approximately 0.18 (g-lactic acid/g-methanol). This remains relatively low compared to reported yields of 0.98 (g-lactic acid/g-sucrose) for L-lactic acid production using *Lactobacillus* spp. (Kotzamanidis et al., 2002) and 0.89 (g-lactic acid/g-glucose) for D-lactic acid production using *S. cerevisiae* (Watcharawipas et al., 2021). It is expected that a rational metabolic engineering strategy based on the findings of this study will lead to further improvements in lactic acid productivity from methanol.

UV mutagenesis has the advantage that it is easy to obtain a variety of mutant strains and that changes in gene expression can be easily investigated by genomic and transcriptomic analyses of mutant strains. These benefits have led to research on UV mutagenesis in various microorganisms. Weng et al. reported that S-adenosyl-L-methionine production in *S. cerevisiae* is associated with enhanced ATP synthesis through the reinforcement of the TCA cycle and gluconeogenesis/glycolysis pathways (Weng et al., 2022). Similarly, Kamba et al. demonstrated that genes related to glyceraldehyde 3-phosphate and dihydroxyacetone phosphate synthesis contributed to improved triacylglycerol production in yeast *Lipomyces starkeyi* (Kamba et al., 2024). If a phenotype can be enhanced by random mutagenesis, such as UV mutagenesis, a detailed analysis of the mutant strains can be used to identify previously unnoticed genes that affect the target phenotype. Furthermore, the identified genes can be manipulated by overexpression or deletion to further enhance the desired phenotype. Even in modern metabolic engineering, where the latest technologies such as genome editing are frequently used, random mutagenesis is still considered useful. In the future, genomic analysis of the obtained mutant strains in this study is expected to be conducted and integrated with transcriptome analysis results, facilitating the development of rational metabolic engineering strategies for the efficient production of valuable compounds from methanol by engineered *K. phaffii*.

GO enrichment analysis of DEGs-UP in the mutant strain DLac_Mut2_221 included genes involved in thiamine synthesis and serine-glycine metabolism (Fig. 6(A)). Thiamine is an essential compound for cellular energy metabolism as its derivatives function as cofactors for various enzymes including transketolase, pyruvate decarboxylase, and oxoglutarate dehydrogenase (Makeeva et al., 2023). The increased expression of thiamine synthesis genes in DLac_Mut2_221 may have enhanced carbon metabolism, including pyruvate metabolism, contributing to improved D-lactic acid productivity. Regarding individual genes related to thiamine synthesis, increased expression of PAS_chr3_0648 (*THI4* (Thiazole synthase)), PAS_chr4_0065 (HMP (Protein involved in synthesis of the thiamine precursor hydroxymethylpyrimidine)), and PAS_chr3_0842 was confirmed (Supplementary Table S1). *THI4* not only promotes thiazole synthesis but also participates in maintaining mitochondrial DNA stability (Medina-Silva et al., 2006). The increased expression of the *THI4* gene may also contribute to improved cellular stress tolerance. Strains lacking *THI4* have been reported to show reduced tolerance to DNA damage caused by UV and EMS (Machado et al., 1997). In this study, the increased expression of *THI4* may have enhanced survival rates following UV-induced mutagenesis. Additionally, the THI4 protein is involved in oxidative and osmotic stress responses (Wolak et al., 2014), suggesting that its increased expression may have improved stress tolerance to methanol in the culture medium and intracellular pH reduction due to D-lactic acid production, thereby enhancing D-lactic acid productivity. Serine is a compound with demand in numerous pathways, including lipid synthesis, redox balance, and bulk protein synthesis (May et al., 2020), and the serine-glycine metabolic pathway in *K. phaffii* is important for formate assimilation (Mitic et al., 2023). Formate has been reported to be assimilable, albeit minimally, using the oxygen tetrahydrofolate pathway and the oxygen-resistant reductive glycine pathway (Mitic et al., 2023). Individual related genes included PAS_chr2-2_0408 (Catabolic L-serine (L-threonine) deaminase) and PAS_chr4_0415 (Cytosolic serine hydroxy methyltransferase), among others (Supplementary Table S1). Serine hydroxy methyltransferase converts glycine to serine, while serine deaminase converts serine to pyruvate. The increased expression of these genes suggests that the supply of serine and pyruvate derived from glycine may have increased within the cells of the DLac_Mut2_221 strain.

**Fig. 8:**
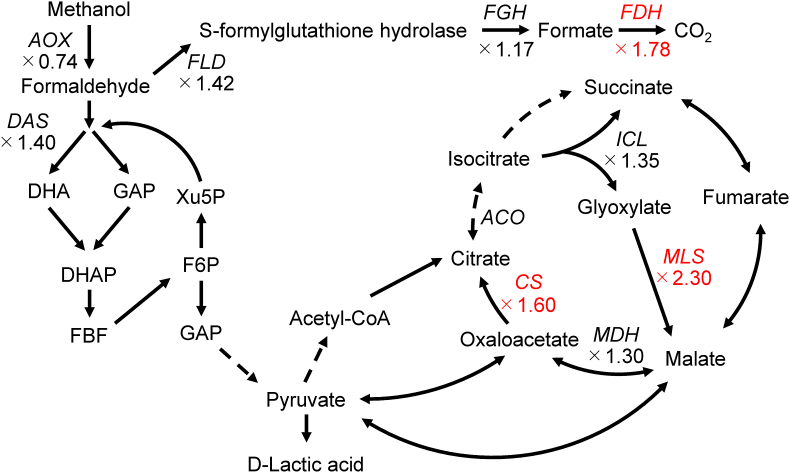
Schematic of methanol utilization pathway in *K. phaffii. ACO*: Aconitase, *AOX*: Alcohol oxidase; *CS*: Citrate synthase; DHA: Dihydroxyacetone, DHAP: Dihydroxyacetone Phosphate, F6P: Fructose-6-phosphate, FBP: Fructose-1,6-Bisphosphate, *FDH*: Formate dehydrogenase; *FGH*: S-formylglutathione hydrolase; *FLD*: Formaldehyde dehydrogenase; GAP: Glyceraldehyde 3-phosphate; *ICL*: Isocitrate lyase; *MDH*: Malate dehydrogenase; *MLS*: Malate synthase; Xu5P: Xylulose 5-phosphate. Solid lines represent a single reaction, whereas dashed lines represent multiple reactions. Enzyme genes marked in red represent upregulated genes.

KEGG pathway enrichment analysis of DEGs-UP in the mutant strain DLac_Mut2_221 included glyoxylate and dicarboxylate metabolism and carbon metabolism (Fig. 7(A)). The glyoxylate cycle consists of five main enzymes: *ICL* (isocitrate lyase), *MLS* (malate synthase), *MDH* (malate dehydrogenase), *CS* (citrate synthase), and *ACO* (aconitase). Although structurally similar to the TCA cycle, it is characterized by the ability to conserve carbon as it does not release CO_2_ during the conversion of isocitrate to succinate and malate by *ICL* and *MLS* (Kunze et al., 2006). In *K. phaffii*, when glucose is used as a carbon source, utilization of the glyoxylate pathway is minimized. However, when methanol is used as a carbon source, the glyoxylate pathway is activated, facilitating the synthesis of C4 compounds (Ruβmayer et al., 2015). Regarding individual genes related to these pathways, increased expression of PAS_chr4_0191 (*MLS*) by 2.30-fold and PAS_chr1-1_0475 (*CS)* by 1.60-fold was confirmed (Supplementary Table S1, Fig. 8). These results suggest activation of the glyoxylate pathway in DLac_Mut2_221. This activation may prevent carbon loss through CO_2_ conversion in the TCA cycle, potentially improving carbon conversion to D-lactic acid. Furthermore, glyoxylate is converted to glycine by *AGTX* (glyoxylate aminotransferase). It is possible that the activation of the glyoxylate pathway in the mutant strain DLac_Mut2_221 increased the supply of glyoxylate, which was then converted to glycine and pyruvate, contributing to improved D-lactic acid production. The enhancement of the carbon metabolism pathway likely indicates improved methanol assimilation ability in the mutant strain DLac_Mut2_221. Improved methanol assimilation may have altered carbon flux for D-lactic acid production. Expression changes were also observed for individual genes included in the carbon metabolism term (Fig. 8). In particular, PAS_chr3_0932 (*FDH* (formate dehydrogenase)) showed a significant increase in expression of 1.78-fold (Supplementary Table S1, Fig. 8). This enzyme is part of the formaldehyde dissimilation pathway (Duman et al., 2020; Yu et al., 2022). In *K. phaffii*, Jia et al. previously discussed formaldehyde toxicity and NADH supply, successfully improving monellin production by suppressing formaldehyde accumulation (Jia et al., 2022). This change in gene expression suggests a potential mechanism where the formaldehyde dissimilation pathways are enhanced, avoiding formaldehyde accumulation. This mechanism may contribute to improved D-lactic acid production from methanol by mitigating formaldehyde-related toxicity.

GO enrichment analysis of DEGs-DOWN primarily contained genes involved in cell wall composition (Fig. 6(B)). These GO terms include genes involved in cell wall synthesis and maintenance, suggesting that the reduced expression of these genes may have caused changes in cell wall composition and structure. Notably, one of the genes with decreased expression is PAS_chr4_0305 (O-glycosylated protein required for cell wall stability (PAS_0305)), which showed a 0.35-fold decrease in expression (Supplementary Table S2). Guo et al. reported that deletion of PAS_0305 increases cell wall permeability, leading to improved protein expression and CMCase activity (Gao et al., 2023). Furthermore, flux balance analysis confirmed that the PAS_0305 deletion strain exhibited reduced carbon loss (Gao et al., 2023). Therefore, the decreased expression of the PAS_0305 gene in the DLac_Mut2_221 strain may have contributed to improved D-lactic acid production through enhanced cell wall permeability and reduced carbon loss. The GO enrichment analysis of the mutant strain DLac_Mut2_221 obtained in this study suggests that genes involved in cell wall composition are important for the production of valuable compounds from methanol using *K. phaffii*. KEGG pathway enrichment analysis of DEGs-DOWN revealed three categories with similar p-values: Cell cycle, Phenylalanine metabolism, and Tyrosine metabolism. These results suggest that the carbon flux and energy balance resulting from methanol uptake shifted toward D-lactic acid production. This may have suppressed cell proliferation and the production of aromatic amino acids (phenylalanine and tyrosine). Indeed, the DLac_Mut2_221 strain showed lower OD_600_ compared to the parent strain and other mutants up to 96 h of cultivation, suggesting that carbon and energy available for cell growth were limited (Fig. 4(B)). This indicates that the carbon source from methanol was primarily directed toward D-lactic acid production, with carbon flux toward cell growth being suppressed.

## 5 Conclusions

In this study, UV mutagenesis of the metabolically engineered D-lactic acid-producing *K. phaffii* GS115/S8/Z3 yielded the mutant strain DLac_Mut2_221, which was capable of efficiently producing D-lactic acid from methanol. The mutant strain was confirmed to produce 5.38 g/L of D-lactic acid from 30 g/L methanol after 168 h of cultivation. To the best of our knowledge, this is the highest concentration of lactic acid, including D- and L-lactic acid, produced from methanol as the sole carbon source. Transcriptome analysis of the mutant strain revealed that three important mechanisms were presumably involved in the increased D-lactic acid production from methanol in the mutant strain: (1) avoiding excessive formaldehyde accumulation, (2) activating the glyoxylate pathway, and (3) reducing the expression of the *O*-glycosylated protein required for cell wall stability. To the best of our knowledge, there are no previous reports linking activation of the glyoxylate pathway or reduced expression of the *O*-glycosylated protein required for cell wall stability to compound production from methanol in *K. phaffii*. This study suggests that these genes may contribute to improving the production of valuable compounds from methanol. Currently, research on the production of valuable compounds from methanol is increasing, and various strategies have been proposed to enhance the yield of target compounds. However, challenges such as low target compound yields and inefficient methanol utilization remain. The insights gained from this study, in combination with previous research on improving compound productivity, will contribute to further enhancing the production efficiency of target compounds from methanol.

## CRediT authorship contribution statement

**Yoshifumi Inoue:** Writing – review & editing, Writing – original draft, Investigation. **Kaito Nakamura:** Investigation. **Ryosuke Yamada:** Writing – review & editing, Writing – original draft, Supervision, Funding acquisition, Conceptualization. **Takuya Matsumoto:** Supervision. **Hiroyasu Ogino:** Supervision.

## Ethics approval and consent to participate

Not applicable.

## Availability of data and materials

The datasets used and/or analyzed in the current study are available from the corresponding author upon reasonable request.

## Funding

This study was partially supported by the 10.13039/501100001691Japan Society for the Promotion of Science (Grant number JP22H03803).

## Declaration of competing interest

The authors declare that they have no known competing financial interests or personal relationships that could have appeared to influence the work reported in this paper.

## Data Availability

Data will be made available on request.
