# Supplementary material for: Improvement of D-lactic acid production from methanol by metabolically engineered Komagataella phaffii via ultra-violet mutagenesis
Source: Metab Eng Commun. 2025 May 17;20:e00262. doi: 10.1016/j.mec.2025.e00262 (PMC12148619; doi:10.1016/j.mec.2025.e00262)
Supplement: Multimedia component 1 [file mmc1.docx]

**Supplementary information**

**Improvement of D-lactic acid production from methanol by metabolically engineered *Komagataella phaffii* via ultra-violet mutagenesis**

Yoshifumi Inoue, Kaito Nakamura, Ryosuke Yamada, Takuya Matsumoto, and Hiroyasu Ogino

**Table S1. Upregulated genes in the mutant strain DLac_Mut2_221**

| Gene product | Locus tag | Fold change | -Log10 (p-value) |
| --- | --- | --- | --- |
| Uncharacterized protein | PAS_chr4_0550 | 2.60 | 40.00 |
| Glycerol kinase, converts glycerol to glycerol-3-phosphate | PAS_chr4_0783 | 2.55 | 40.00 |
| Putative channel-like protein | PAS_chr3_0648 | 2.35 | 40.00 |
| Thiazole synthase, catalyzes formation of the thiazole moiety of thiamine pyrophosphate | PAS_chr4_0784 | 2.35 | 40.00 |
| Malate synthase | PAS_chr4_0191 | 2.30 | 40.00 |
| Protein involved in synthesis of the thiamine precursor hydroxymethylpyrimidine (HMP) | PAS_chr1-1_0158 | 2.17 | 40.00 |
| Putative transmembrane protein involved in export of ammonia, a starvation signal | PAS_chr2-2_0492 | 2.17 | 40.00 |
| Uncharacterized protein | PAS_chr4_0065 | 2.17 | 40.00 |
| Nuclear protein related to mammalian high mobility group (HMG) proteins | PAS_chr4_0796 | 2.14 | 40.00 |
| Acetate--CoA ligase | PAS_chr3_0389 | 2.01 | 7.66 |
| Hypothetical protein | PAS_chr2-1_0767 | 2.01 | 40.00 |
| Hypothetical protein | PAS_chr4_0838 | 2.01 | 40.00 |
| Uncharacterized protein | PAS_chr2-2_0482 | 2.00 | 4.11 |
| Hypothetical protein | PAS_chr1-4_0481 | 1.97 | 40.00 |
| Ubiquitin | PAS_chr4_0762 | 1.92 | 40.00 |
| Cytochrome c, isoform 1 | PAS_chr4_0274 | 1.85 | 10.06 |
| Essential nucleolar protein involved in the early steps of 35S rRNA processing | PAS_chr4_0018 | 1.85 | 40.00 |
| Uncharacterized protein | PAS_chr4_0438 | 1.84 | 22.17 |
| Hypothetical protein | PAS_chr2-1_0088 | 1.83 | 8.63 |
| Hypothetical protein | PAS_chr4_0381 | 1.82 | 14.46 |
| Uncharacterized protein | PAS_chr3_0362 | 1.82 | 40.00 |
| Hexokinase-2 | PAS_chr4_0155 | 1.80 | 17.59 |
| Hypothetical protein | PAS_chr1-4_0561 | 1.80 | 40.00 |
| Hypothetical protein | PAS_chr2-1_0353 | 1.80 | 40.00 |
| Thioredoxin peroxidase, acts as both a ribosome-associated and free cytoplasmic antioxidant | PAS_chr2-2_0220 | 1.80 | 40.00 |
| H subunit of the mitochondrial glycine decarboxylase complex | PAS_chr4_0627 | 1.79 | 40.00 |
| Plasma membrane localized protein that protects membranes from desiccation | PAS_FragB_0009 | 1.79 | 40.00 |
| Hypothetical protein | PAS_chr3_0932 | 1.78 | 40.00 |
| Mitochondrial 54S ribosomal protein YmL39 | PAS_chr3_1135 | 1.78 | 40.00 |
| NAD(+)-dependent formate dehydrogenase, may protect cells from exogenous formate | PAS_chr4_0781 | 1.78 | 40.00 |
| Fe(II)-dependent sulfonate/alpha-ketoglutarate dioxygenase, involved in sulfonate catabolism for use | PAS_chr2-1_0198 | 1.77 | 9.93 |
| Putative acyltransferase with similarity to Eeb1p and Eht1p | PAS_chr4_0086 | 1.77 | 16.80 |
| putative protein kinase, overexpression causes sensitivity to staurosporine | PAS_chr4_0666 | 1.75 | 10.27 |
| Uncharacterized protein | PAS_chr4_0407 | 1.75 | 40.00 |
| Uncharacterized protein | PAS_chr4_0715 | 1.74 | 10.18 |
| Uncharacterized protein | PAS_chr4_0439 | 1.74 | 22.23 |
| Uncharacterized protein | PAS_chr4_0567 | 1.74 | 40.00 |
| Fe(II)-dependent sulfonate/alpha-ketoglutarate dioxygenase, involved in sulfonate catabolism for use | PAS_chr2-1_0014 | 1.72 | 25.75 |
| Putative transmembrane protein involved in export of ammonia | PAS_chr1-1_0378 | 1.72 | 40.00 |
| Hypothetical protein | PAS_chr4_0683 | 1.71 | 40.00 |
| Constituent of the mitochondrial import motor associated with the presequence translocase | PAS_chr1-3_0038 | 1.69 | 24.30 |
| Hypothetical protein | PAS_chr4_0309 | 1.67 | 4.81 |
| Hypothetical protein | PAS_chr2-1_0235 | 1.67 | 40.00 |
| Na+/Pi cotransporter, active in early growth phase | PAS_chr4_0080 | 1.67 | 40.00 |
| Hypothetical protein | PAS_chr2-2_0403 | 1.66 | 20.37 |
| Uncharacterized protein | PAS_chr4_0271 | 1.66 | 22.68 |
| Uncharacterized protein | PAS_chr1-4_0678 | 1.66 | 25.27 |
| E3 ubiquitin ligase for Rad6p | PAS_chr4_0804 | 1.65 | 9.39 |
| Hypothetical protein | PAS_chr3_0842 | 1.65 | 10.98 |
| Hypothetical protein | PAS_chr4_0172 | 1.65 | 12.80 |
| Peroxisomal 2,4-dienoyl-CoA reductase, auxiliaryenzyme of fatty acid beta-oxidation | PAS_chr2-1_0748 | 1.65 | 40.00 |
| Subunit of the prohibitin complex (Phb1p-Phb2p) | PAS_chr2-2_0019 | 1.65 | 40.00 |
| Uncharacterized protein | PAS_chr4_0505 | 1.65 | 40.00 |
| Hypothetical protein | PAS_chr1-4_0560 | 1.64 | 40.00 |
| Mitochondrial alcohol dehydrogenase isozyme III | PAS_chr2-1_0472 | 1.64 | 40.00 |
| Uncharacterized protein | PAS_chr2-1_0853 | 1.64 | 40.00 |
| Uncharacterized protein | PAS_chr4_0704 | 1.64 | 40.00 |
| ATPase involved in protein import into the ER, also acts as a chaperone to mediate protein folding i | PAS_chr1-4_0630 | 1.62 | 14.02 |
| Protein containing an N-terminal epsin-like domain | PAS_chr4_0551 | 1.62 | 20.17 |
| Putative S-adenosylmethionine-dependent methyltransferase of the seven beta-strand family | PAS_chr4_0763 | 1.62 | 22.40 |
| Uncharacterized protein | PAS_chr2-1_0140 | 1.62 | 40.00 |
| ATPase involved in protein folding and the response to stress | PAS_chr1-1_0023 | 1.61 | 21.38 |
| Hypothetical protein | PAS_chr3_0959 | 1.61 | 23.90 |
| Mitochondrial 54S ribosomal protein YmL38/YmL34 | PAS_chr3_0230 | 1.61 | 40.00 |
| Mitochondrial aldehyde dehydrogenase | PAS_chr1-1_0475 | 1.60 | 40.00 |
| Mitochondrial cytochrome-c peroxidase | PAS_chr2-2_0127 | 1.60 | 40.00 |
| Uncharacterized protein | PAS_chr4_0043 | 1.60 | 40.00 |
| 60S ribosomal protein L22 | PAS_chr4_0944 | 1.59 | 19.25 |
| Uncharacterized protein | PAS_chr4_0041 | 1.59 | 40.00 |
| Hypothetical protein | PAS_chr4_0782 | 1.58 | 5.48 |
| Hypothetical protein | PAS_chr4_0775 | 1.58 | 7.36 |
| Uncharacterized protein | PAS_chr3_0525 | 1.58 | 15.79 |
| Alpha 3 subunit of the 20S proteasome, the only nonessential 20S subunit | PAS_chr4_0388 | 1.57 | 2.97 |
| Endoplasmic reticulum (ER) resident protein | PAS_chr4_0250 | 1.57 | 4.46 |
| Hypothetical protein | PAS_chr4_0570 | 1.57 | 8.71 |
| Mitochondrial ribosomal protein of the large subunit | PAS_chr4_0535 | 1.57 | 14.07 |
| Uncharacterized protein | PAS_chr4_0506 | 1.57 | 40.00 |
| Beta-tubulin | PAS_chr4_0916 | 1.56 | 5.63 |
| Essential protein, component of a complex containing Cef1p | PAS_chr1-4_0411 | 1.56 | 9.19 |
| Evolutionarily conserved subunit of the CCR4-NOTcomplex involved in controlling mRNA initiation | PAS_chr4_0224 | 1.56 | 14.61 |
| Uncharacterized protein | PAS_chr4_0533 | 1.56 | 25.34 |
| Catalytic subunit of N-terminal acetyltransferase of the NatC type | PAS_chr4_0541 | 1.55 | 4.77 |
| Protein required for respiratory growth and stability of the mitochondrial genome | PAS_chr2-1_0189 | 1.55 | 16.69 |
| Protein that forms a complex with the Sit4p protein phosphatase and is required for its function | PAS_chr3_0234 | 1.55 | 17.58 |
| Uncharacterized protein | PAS_chr3_1230 | 1.55 | 40.00 |
| Catabolic L-serine (L-threonine) deaminase, catalyzes the degradation of L-serine and L-threonine | PAS_chr2-2_0408 | 1.54 | 10.96 |
| Cytosolic serine hydroxymethyltransferase | PAS_chr4_0869 | 1.54 | 11.33 |
| Non-essential protein of unknown function | PAS_chr4_0415 | 1.54 | 40.00 |
| Cytoplasmic thioredoxin isoenzyme of the thioredoxin system | PAS_chr1-3_0112 | 1.53 | 5.50 |
| Putative RNA-binding protein implicated in ribosome biogenesis | PAS_chr1-4_0478 | 1.53 | 18.23 |
| Regulatory, non-ATPase subunit of the 26S proteasome | PAS_chr4_0284 | 1.53 | 40.00 |
| Acireductone dioxygenease involved in the methionine salvage pathway | PAS_chr1-3_0124 | 1.52 | 3.31 |
| Hypothetical protein | PAS_chr2-1_0422 | 1.52 | 14.13 |
| Mitochondrial 54S ribosomal protein YmL44 | PAS_chr4_0572 | 1.52 | 40.00 |
| Hypothetical protein | PAS_chr4_0281 | 1.51 | 3.65 |
| Hypothetical protein | PAS_chr3_0884 | 1.51 | 10.78 |
| Hypothetical protein | PAS_chr2-1_0337 | 1.51 | 11.88 |
| Hypothetical protein | PAS_chr3_0401 | 1.51 | 15.30 |
| Subunit of a heterodimeric NC2 transcription regulator complex with Bur6p | PAS_chr3_0261 | 1.51 | 23.58 |

**Table S2**. **Downregulated genes in the mutant strain DLac_Mut2_221**

| Gene product | Locus tag | Fold change | -Log10 (p-value) |
| --- | --- | --- | --- |
| Hypothetical protein | PAS_chr4_0151 | 0.11 | 40.00 |
| Hypothetical protein | PAS_chr2-1_0002 | 0.33 | 40.00 |
| C-8 sterol isomerase | PAS_chr4_0198 | 0.37 | 40.00 |
| uncharacterized protein | PAS_chr4_0820 | 0.43 | 40.00 |
| Putative chitin transglycosidase, cell wall protein | PAS_chr4_0559 | 0.46 | 40.00 |
| Mitochondrial outer membrane and cell wall localized SUN family member | PAS_chr4_0046 | 0.47 | 40.00 |
| Uncharacterized protein | PAS_chr4_0914 | 0.47 | 40.00 |
| Pho85 cyclin of the Pcl1,2-like subfamily, involved in entry into the mitotic cell cycle and regulat | PAS_chr2-1_0092 | 0.49 | 18.15 |
| Uncharacterized protein | PAS_chr4_0926 | 0.51 | 40.00 |
| Uncharacterized protein | PAS_chr2-2_0489 | 0.51 | 40.00 |
| Ammonium permease involved in regulation of pseudohyphal growth | PAS_chr1-4_0394 | 0.53 | 11.35 |
| Hypothetical protein | PAS_chr4_0461 | 0.53 | 12.21 |
| O-glycosylated protein required for cell wall stability | PAS_chr4_0305 | 0.53 | 40.00 |
| Uncharacterized protein | PAS_chr1-1_0479 | 0.54 | 40.00 |
| Aromatic aminotransferase II | PAS_chr4_0147 | 0.54 | 40.00 |
| Hypothetical protein | PAS_chr1-4_0327 | 0.55 | 40.00 |
| Uncharacterized protein | PAS_chr1-1_0482 | 0.55 | 40.00 |
| Hypothetical protein | PAS_chr4_0089 | 0.55 | 11.56 |
| 60S ribosomal protein L22 | PAS_chr4_0041 | 0.58 | 40.00 |
| Hypothetical protein | PAS_chr4_0114 | 0.58 | 17.63 |
| Major exo-1,3-beta-glucanase of the cell wall, involved in cell wall beta-glucan assembly | PAS_chr2-1_0454 | 0.58 | 15.28 |
| Hypothetical protein | PAS_chr1-4_0554 | 0.58 | 3.87 |
| Hypothetical protein | PAS_chr1-1_0234 | 0.58 | 21.63 |
| Uncharacterized protein | PAS_chr2-2_0488 | 0.58 | 40.00 |
| Mitochondrial intermembrane space protein, formsa complex with TIm8p | PAS_chr4_0561 | 0.59 | 8.20 |
| Uncharacterized protein | PAS_chr1-3_0290 | 0.59 | 8.68 |
| Vacuolar cation channel | PAS_chr4_0220 | 0.59 | 11.46 |
| Protoporphyrinogen oxidase | PAS_chr4_0055 | 0.59 | 12.71 |
| Uncharacterized protein | PAS_chr4_0460 | 0.60 | 5.47 |
| 40S ribosomal protein S6 | PAS_chr4_0094 | 0.61 | 40.00 |
| Hypothetical protein | PAS_chr4_0335 | 0.61 | 11.58 |
| Uncharacterized protein | PAS_chr4_0779 | 0.61 | 4.54 |
| Uncharacterized protein | PAS_FragB_0059 | 0.61 | 5.64 |
| ADP/ATP carrier protein | PAS_chr4_0210 | 0.61 | 40.00 |
| Cytoplasmic RNA-binding protein, contains an RNArecognition motif (RRM) | PAS_chr2-1_0414 | 0.61 | 7.88 |
| Hypothetical protein | PAS_chr1-4_0583 | 0.61 | 2.26 |
| Hypothetical protein | PAS_chr4_0628 | 0.62 | 21.30 |
| Hypothetical protein | PAS_chr4_0152 | 0.62 | 40.00 |
| Uncharacterized protein | PAS_chr1-3_0303 | 0.62 | 8.74 |
| Hypothetical protein | PAS_chr4_0590 | 0.63 | 16.26 |
| Uncharacterized protein | PAS_chr4_0972 | 0.63 | 40.00 |
| Bud neck-localized, SH3 domain-containing protein required for cytokinesis | PAS_chr2-2_0259 | 0.64 | 9.04 |
| Hypothetical protein | PAS_chr1-4_0240 | 0.64 | 3.85 |
| Hypothetical protein | PAS_chr4_0553 | 0.64 | 10.83 |
| Uncharacterized protein | PAS_chr3_0076 | 0.64 | 40.00 |
| Hypothetical protein | PAS_chr2-2_0117 | 0.64 | 10.98 |
| Hypothetical protein | PAS_chr4_0883 | 0.64 | 40.00 |
| Polyamine transport protein specific for spermine | PAS_chr1-3_0215 | 0.64 | 11.92 |
| Vacuolar carboxypeptidase yscS | PAS_chr4_0686 | 0.64 | 40.00 |
| Putative dihydrokaempferol 4-reductase | PAS_chr4_0336 | 0.65 | 14.19 |
| Uncharacterized protein | PAS_chr4_0919 | 0.65 | 11.60 |
| G1/S-specific cyclin | PAS_FragB_0046 | 0.66 | 4.52 |
| L-homoserine-O-acetyltransferase, catalyzes the conversion of homoserine to O-acetyl homoserine | PAS_chr2-1_0383 | 0.66 | 6.96 |
| Meiosis-specific protein of unknown function, required for spore wall formation during sporulation | PAS_chr4_0560 | 0.66 | 5.54 |
| Uncharacterized protein | PAS_chr1-3_0299 | 0.66 | 7.27 |
| Uncharacterized protein | PAS_chr4_0915 | 0.66 | 8.09 |
| Uncharacterized protein | PAS_chr3_1239 | 0.66 | 2.02 |
| Uncharacterized protein | PAS_chr2-1_0887 | 0.66 | 40.00 |
| Protein of the SUN family (Sim1p, Uth1p, Nca3p, Sun4p) that may participate in DNA replication | PAS_chr2-2_0064 | 0.67 | 27.65 |
